# Synthesis of a Versatile Building Block Combining Cyclen-derivative DO3A with a Polyamine via a Rigid Spacer

**DOI:** 10.3390/molecules181113940

**Published:** 2013-11-12

**Authors:** Bohuslav Drahoš, Zdeněk Trávníček

**Affiliations:** Department of Inorganic Chemistry & Regional Centre of Advanced Technologies and Materials, Faculty of Science, Palacký University, 17. listopadu 12, Olomouc CZ-77146, Czech Republic; E-Mail: bohuslav.drahos@upol.cz

**Keywords:** synthesis, DO3A macrocycle, building block, NMR spectroscopy, mass spectrometry

## Abstract

The five-step synthesis of a polydentate building block combining a cyclen-based macrocycle (DO3A) with *N*-(2-aminoethyl)propane-1,3-diamine, which are linked through the xylylen moiety as a rigid C-spacer is described. These two molecular parts were coupled by subsequent bromine atom substitution in 1,4-bis(bromomethyl)benzene. First, *N*-(2-aminoethyl)propane-1,3-diamine was protected by phthaloyl moieties and then it was reacted with 1,4-bis(bromomethyl)benzene to form (2-phthalimidoethyl)(3-phthalimido-prop-1-yl)(4-bromomethylbenzyl)amine (**2**). This compound underwent a substitution reaction with DO3A in the form of its *tert*-butyl esters leading to the intermediate 1-{4-[(2-phthalimidoethyl)(3-phthalimidoprop-1-yl)aminomethyl]phenylmethyl}-4,7,10-tris(*t*-butoxy-carbonylmethyl)-1,4,7,10-tetraazacyclododecane (**3**). The phthaloyl as well as the *t*-butyl protecting groups were removed in the next two reaction steps to form the final product 1-{4-[(2-aminoethyl)(3-aminoprop-1-yl)aminomethyl]phenylmethyl}-4,7,10-tris(carboxy-methyl)-1,4,7,10-tetraazacyclododecane (**5**). The intermediates **1**–**4** as well as the final product **5** were characterized by elemental analysis, mass spectrometry, and multinuclear (^1^H and ^13^C) and two-dimensional NMR spectroscopy. The final product **5** could serve as a potential building block in subsequent syntheses of binuclear complexes of lanthanides and/or transition metals.

## 1. Introduction

**Figure 1:**
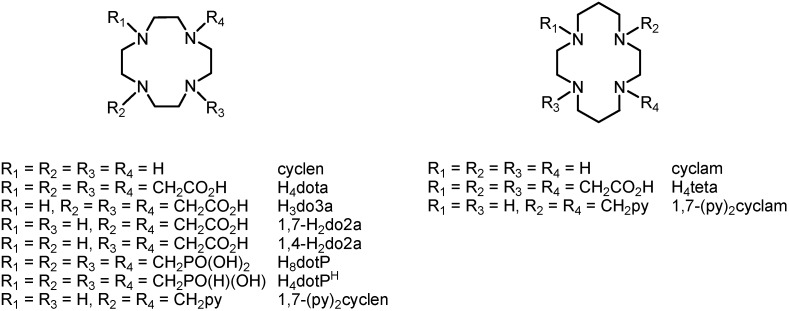
Structural representations and abbreviations of selected macrocycles derived from cyclen (*left*) and cyclam (*right*).

Polyazamacrocycles have become a large and variable group of important organic substances because they represent irreplaceable ligands in complexes with transition metals, lanthanides, actinides or they are chemical and structural bases of different anion receptors. One group of the most common examples involves tetraazamacrocycles, namely cyclen (1,4,7,10-tetraazacyclododecane) and cyclam (1,5,8,11-tetraazacyclotetradecane), which have found many applications as contrast agents for Magnetic Resonance Imaging (MRI) [1], in optical imaging [2], Positron Emission Tomography (PET) [3], Single-Photon Emission Computed Tomography (SPECT) [3] and as radiotherapeutics [4] as well. Many structurally modified analogues have been prepared (representative examples are shown in Figure 1), such as the well-known *N*-substituted derivatives with a different number of pendant arms containing carboxylic acids (H_4_dota 1,4,7,10-tetraazacyclododecane-1,4,7,10-tetraacetic acid, H_3_do3a 1,4,7,10-tetraazacyclododecane-1,4,7-triacetic acid, H_2_do2a 1,4,7,10-tetraazacyclododecane-1,4- or -1,7-dicarboxylic acid, H_4_teta 1,4,8,11-tetraazacyclotetradecane-1,4,8,11-tetraacetic acid, and many others), phosphonic acid (H_8_dotP 1,4,7,10-tetraazacyclododecane-1,4,7,10-tetrakis(methylphosphonic acid) *etc.*) or phosphinic acid (H_4_dotP^H^ 1,4,7,10-tetraazacyclododecane-1,4,7,10-tetra-kis(methylphosphinic acid)) [1]. Gd(III) complexes with these compounds have found a unique application as contrast agents for MRI, ([Gd(dota)H_2_O]^−^ is one of the most stable and inert gadolinium complexes known under the trade name DOTAREM^®^), and complexes of other lanthanides are employed in luminescence imaging and assay, or their complexes with ^64/67^Cu serve as PET agents.

**Figure 2:**
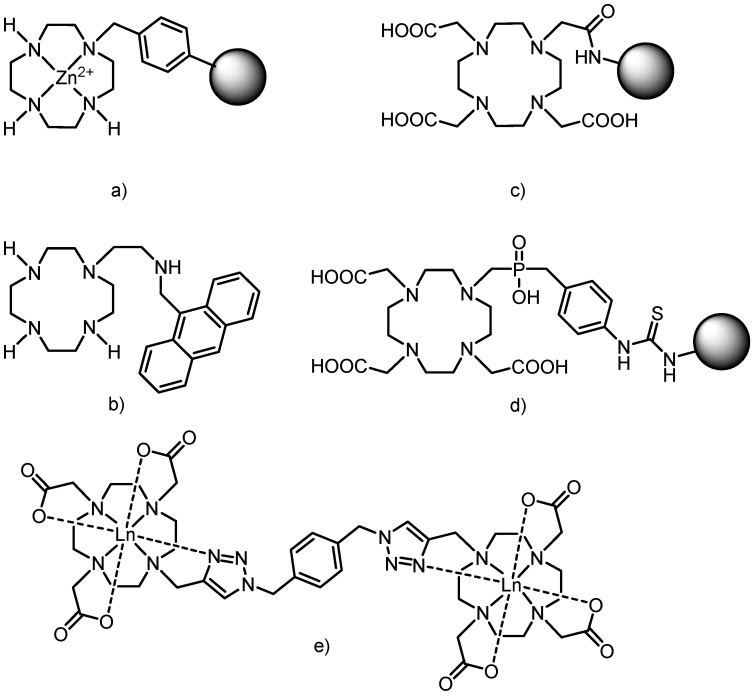
Examples of structures combining macrocycles with each other or with other molecules of interest. (**a**) Zn(II)-cyclen part connected to a polymer (represented as a sphere). (**b**) Cyclen modified with anthrylmethylamino group. (**c**) DO3A moiety connected by the amide bridge to G0–G5 poly(amidoamine) (PAMAM) dendrimers (represented as a sphere). (**d**) Thiourea bridge between the macrocycle and G2 PAMAM dendrimer (represented as a sphere). (**e**) Example of two macrocycles connected via “click chemistry” reaction (copper catalyzed [3+2] cycloaddition between alkyne and organic azide) and the mode of lanthanide coordination.

Surprisingly, not much attention has been devoted to the characterization of the magnetic properties of solid metal complexes with this type of macrocyclic ligands, except for a series of Fe(II) complexes with cyclen/cyclam modified on nitrogen atoms with different alkyls or pyridine-containing pendant arms (1,7-(py)_2_cyclen or 1,7-(py)_2_cyclam), e.g., [Fe(1,7-(py)_2_cyclam)](BF_4_)_2_·H_2_O showed spin-crossover behavior [5]. Additional progress in cyclen/cyclam-based chemistry was achieved by connecting the macrocycles, usually by a rigid linker, to produce receptors for different anions (e.g., phosphates recognized by a Zn(II) complex of cyclen anchored onto a polymer, Figure 2a) [6], cations (Cu(II) or Zn(II) receptors having cyclen attached to a chromophore—dansyl (5-(dimethyl-amino)naphthalene-1-sulfonyl), or anthryl group, Figure 2b) or neutral molecules (riboflavin, creatinine or thymine) [7].

Further improvement, especially in the field of MRI contrast agents and radiotherapeutics, was achieved by linking the macrocycles with another molecule of interest. Gd(III) complexes of macrocycles linked to the macromolecules (dendrimers, cyclodextrins, proteins, nanotubes, *etc.*) showed improved efficiency as MRI contrast agents (slowing down molecular rotation and tumbling) [1]. The second group of molecules, which were linked to the macrocycle, could be considered as biological targets and provided specific delivery or metabolic pathway of the molecule in living organisms [8]. The third group is comprised of molecules which showed a specific function and/or property, and when they were combined with the macrocycle, new classes of multimodal/multifunctional compounds were obtained (MRI and near-infrared (NIR) contrast agents, MRI and PET agents, “theranostic” agents combining contrast agent and therapeutics into one molecule, *etc.*) [9].

The DO3A moiety has been widely used for the above-mentioned linking because it contains only one site for possible substitution. Many different spacers between the macrocycle and the other molecule of interest have been used and they are mostly based on the amide bond (Figure 2c) [10], thiourea bridge (Figure 2d) [11] and different types of “click chemistry” bonding (e.g., particularly copper catalyzed [3+2] cycloaddition reactions between alkynes and organic azides, Figure 2e) [12]. Here it should be stated that only representative examples of each group of compounds are mentioned because their complete lists are well beyond the scope of this Introduction and they can be found in many monographs and reviews [1,13].

**Figure 3:**
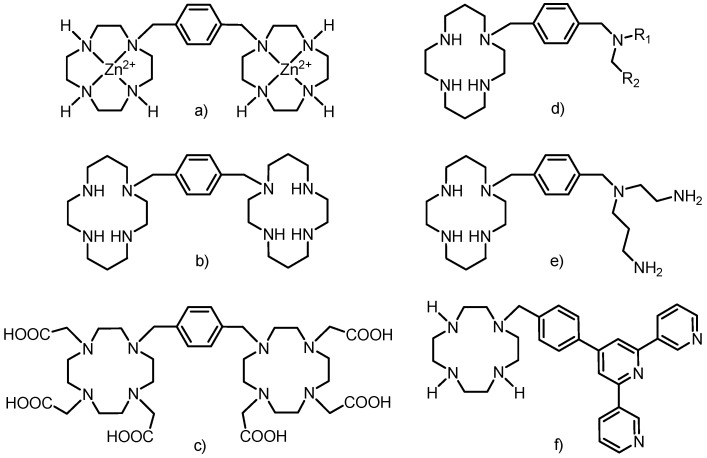
Schematic representations of compounds in which cyclen/cyclam (or its derivatives) were connected to polyamine via the xylylen bridge. For (d) R_1_ = H or Me, R_2_ = 2-, 3- or 4-pyridine or 2-aminophenyl, 4-aminophenyl, 5-methylpiperazine.

The rigid xylylen (1,4-dimethylbenzene-1,4-diyl) bridge has already been employed as a linker between the macrocyle and polyamine, but not exactly in the way described in this work. Two cyclen or cyclam molecules were linked by the xylylen bridge in the synthesis of selective anti-HIV-1 agents (as Zn(II) complexes or free amines, Figure 3a,b) [7] and previously discussed anion receptors [6]. Two DO3A macrocycles were already connected into one molecule by the xylylen bridge (Figure 3c) [14] and its lanthanide complexes showed enhanced luminescence due to the presence of the benzene ring (xylylen) which acted as a sensitizing chromophore and caused efficient energy transfer (an antenna effect) [14].

Structurally similar compounds to that studied in this paper were prepared by connection of cyclen or cyclam with linear polyamines. The anti-HIV-1 activity was described for monosubstituted cyclam containing different monoamines modified by pyridine or aminophenyl groups (Figure 3d) [15] as well as cyclam substituted with *N*-(2-aminoethyl)propane-1,3-diamine (Figure 3e) [16,17]. The synthesis of the latter used phthaoyl protecting groups, but in comparison with our work, the further modification of the macrocycle with acetate pendant arms was not persued.

The synthetic approach used for the preparation of the above-mentioned ligands was based on the reaction of protected cyclen/cyclams with 1,4-bis(bromomethyl)benzene (one bromine atom is substituted) and the obtained monobrominated intermediate reacted with differently protected molecules (other cyclen/cyclam, linear polyamines, *etc.*). The terpyridine moiety was connected to DO3A via a structurally similar rigid phenylmethyl bridge (Figure 3f) to obtain a self-assembly heterotrinuclear gadolinium(III)–iron(II) complex which belongs to a new class of MRI contrast agents called metallostars [18,19].

In this paper, the synthesis of a polydentate polyamino-polyacetate 1-{4-[(2-aminoethyl)(3-aminoprop-1-yl)aminomethyl]phenylmethyl}-4,7,10-tris(carboxymethyl)-1,4,7,10-tetraazacyclododecane (**5**) combining DO3A with *N*-(2-aminoethyl)propane-1,3-diamine connected with the rigid xylylen bridge is described. All synthetic steps are described in detail and compared with similar previously published procedures. This type of molecule has been chosen because it provides a good starting point for the synthesis of more sophisticated ligands belonging to the above-mentioned group of multimodal/multifunctional compounds. The synthetically readily available DO3A cavity is a selective chelator of lanthanides (providing e.g., luminescence) and the polyamine part can complex different transition metals (providing e.g., spin-crossover behavior). Additionally, the polyamine part of the molecule is simply modifiable in a way necessary for required complexation properties. The xylylen linker has been chosen because it is relatively rigid and short enough, and moreover, it involves a delocalized electron density, which all together may be a good condition for the preparation of complexes showing interesting magnetic properties in the case of incorporation of two suitable transition metals into the structure of **5**. Moreover, despite a large number of structurally similar compounds, to the best of our knowledge, no such motif of the rigid C-bonded (xylylen) bridge between DO3A and a linear polyamine can be found in the literature so far.

## 2. Results and Discussion

**Scheme 1:**
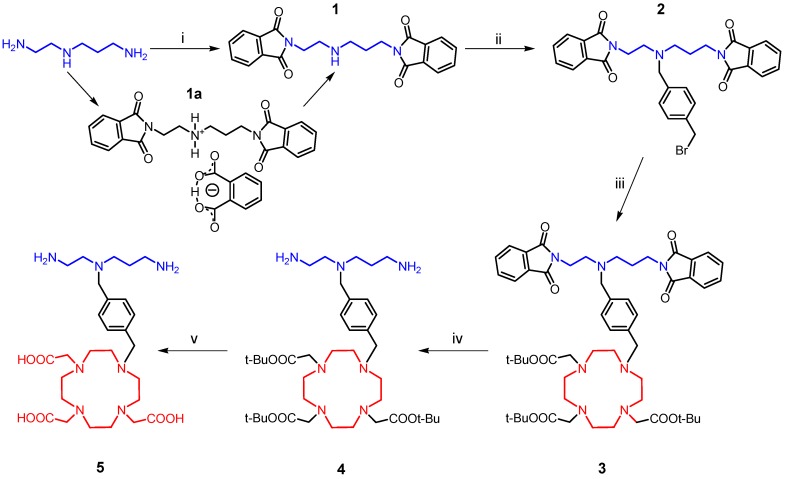
The synthetic pathway leading to the final product **5**.

**Figure 4:**
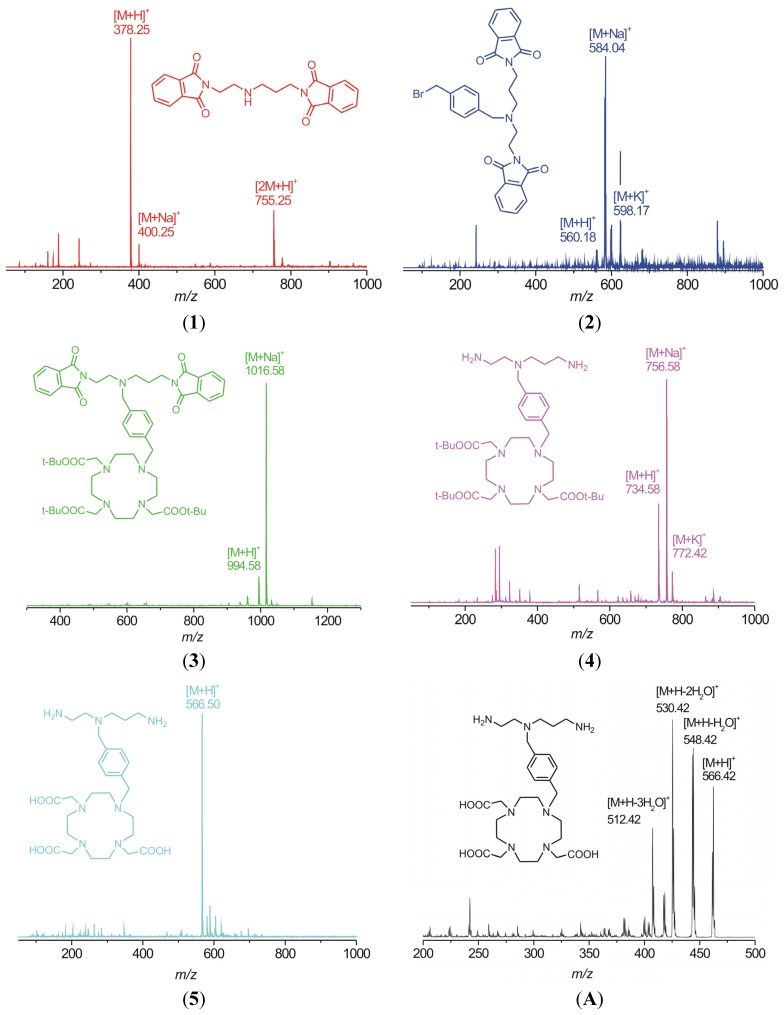
ESI mass spectra of intermediates **1**–**4** and the final product **5** showing the molecular peaks (adducts with H^+^, Na^+^ or K^+^) together with MS^2^ pattern of the molecular ion *m/z* 566.50 (**A**).

**Figure 5:**
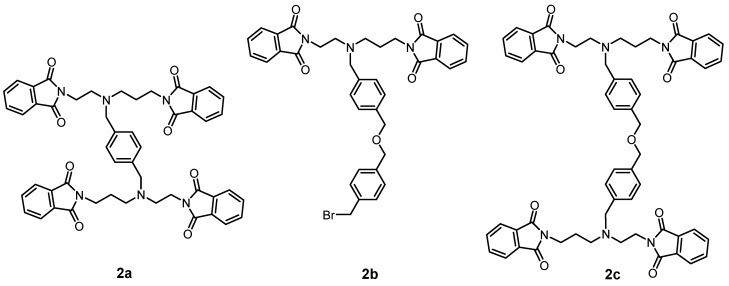
Suggested structures of species identified in the mass spectrum of the reaction mixture during the preparation of **2**. *m/z* values for **2a**: 856.25 [M+H]^+^, **2b**: 681.17 [M+H]^+^ and **2c**: 977.17 [M+H]^+^.

The synthesis of the desired polydentate molecule **5** was done in five steps which are outlined in Scheme 1 and the corresponding electrospray ionization (ESI) mass spectra are depicted in Figure 4. The well-known phthalimide protection was employed in the first step of the synthesis. The intermediate **1** was prepared in a good yield of 69% according to the procedure described in [20,21] with a little modification. Glacial acetic acid was used as a solvent and in this case, the product, which precipitated from the ethanolic (EtOH) solution, was slightly contaminated with diphthalimidodiethylammonium hydrogen phthalate (**1a**, see Experimental section) [21]. The deprotonation of **1a**, leading to **1**, and additional removal of the hydrogen phthalate anion were done by treatment with 10% aqueous solution of Na_2_CO_3_. This step was necessary for the removal of the prevailing traces of acetic acid as well. The similar reaction using acetonitrile (MeCN) as a solvent, described in [22] was unsuccessful, probably due to the low solubility of phthalic acid anhydride in MeCN. In the second step, the (4-bromomethyl)benzyl group was incorporated into the molecule of **1** to produce the intermediate **2**. The treatment of **1** with an excess of 1,4-bis(bromomethyl)benzene in refluxing MeCN with K_2_CO_3_ gave **2** in a moderate yield of 51%. The reaction was monitored by thin layer chromatography (TLC) and was completed in 30 min after dropwise addition of **1** to the refluxing mixture of 1,4-bis(bromomethyl)benzene and K_2_CO_3_. The main intermediate **2** was separated from the unreacted 1,4-bis(bromomethyl)benzene and unwanted by-product **2a** (Figure 5) by means of silica gel column chromatography (dichloromethane (DCHM): diethylether from 10:0 to 10:1). To eliminate the formation of the by-product **2a**, the reactants, *i.e.*, **1** and 1,4-bis(bromomethyl)benzene, should be mixed in the molar ratio of 1:2. Additionally, the mass spectra analysis revealed the presence of polymeric substances **2b** and **2c** depicted in Figure 5, which could be related to the utilization of non-dried solvents.

*Reagents and Conditions*: (i) phthalic acid anhydride, CH_3_COOH, reflux 1 h; (ii) 1,4-bis(bromomethyl)benzene, K_2_CO_3_, MeCN, reflux 0.5 h; (iii) DO3A-tris(*t*-butyl ester), K_2_CO_3_, MeCN, reflux 4 h; (iv) hydrazine, EtOH, 25 °C, 12 h; (v) trifluoroacetic acid, dichloromethane, 25 °C, 12 h.

In the next step, the intermediate **2** was coupled with DO3A in the protected form of *t*-butyl esters. The reaction conditions were similar as for the synthesis of **2**, but the reactants were mixed almost immediately. The bromine atom substitution was completed after four hours of reflux resulting in the desired product **3** (see Scheme 1). The crude product was purified by silica gel column chromatography (CHCl_3_–MeOH ≈ 25:1).

Further reaction steps involved the deprotection of the amino and carboxylate groups. Firstly, the elimination of the phthaloyl protection group was performed by the reaction with an excess of hydrazine in EtOH according to the literature procedure [23]. After stirring of this solution at room temperature for 12 h, precipitated phthalhydrazide was filtered off and the intermediate **4** was obtained after the evaporation of the solvent *in vacuo*. The rest of the phthalhydrazide present was removed by precipitation and filtration from the CHCl_3_ solution of the residue.

To deprotect the carboxylic groups on the macrocycle, a familiar reaction with trifluoroacetic acid (TFA) in DCHM was used [24]. Overnight stirring at room temperature was satisfactory, because no signal of *t*-butyl groups was observed in the ^1^H-NMR spectrum of the product obtained after the evaporation of all solvents. In the end, the free amino-carboxylate was purified by cation-exchange and then anion-exchange chromatography. The presence of the carboxylic groups was confirmed by the fragmentation pattern of the molecular ion *m/z* 566.50 in the MS^2^ spectrum (Figure 4) which consists of peaks with the *m/z* = 18 difference which corresponds to the elimination of one, two or three water molecules from one, two or three carboxylates, respectively, in the molecule.

**Figure 6:**
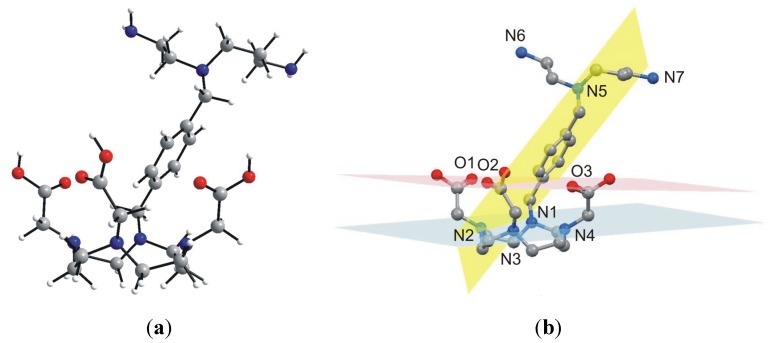
Geometry of **5** optimized at the B3LYB/6-311G * level of theory (*left*, **a**), showing selected least-square planes (*right*, **b**) going through the macrocyclic nitrogen atoms N1–N4 (blue), the carboxylic oxygen atoms O1–O3 (red) and the benzene ring (yellow). Hydrogen atoms are omitted for clarity (b).

With the aim to ambiguously characterize the composition and structure of the final product **5**, many attempts to prepare single-crystals suitable for single-crystal X-ray analysis were done, however, all without success. That is why we decided to optimize the geometry of **5** by means of theoretical calculations. Thus, the geometry of **5** was optimized at the density functional level of theory (DFT), using the B3LYB functional with the 6-311G * basis set. The optimized structure of **5** is depicted in Figure 6. The macrocyclic part of **5** has a cage-like structure with four nitrogen atoms, N1–N4, forming the basal plane (in blue colour in Figure 6) and three oxygen atoms forming the capping plane (in red colour in Figure 6). These two planes are nearly coplanar, forming a dihedral angle of 4.7°. The xylylen spacer with the polyamine part (N5–N7) is situated along the three acetic acid pendant arms and the dihedral angle between the plane formed by its benzene ring (in yellow colour in Figure 6) and basal, and capping plane is 57.7°, and 69.2°, respectively. As for the optimized geometry structure of **5**, the bond lengths and angles of the main individual parts are in good agreement with those determined for DOTA by single-crystal X-ray analysis [25], but only the macrocyclic cavity is slightly expanded (interatomic distances for N1–N3 and N2–N4 are 5.092 Å, and 4.634 Å, respectively). However, we are aware of the fact that the calculations were performed in vacuum and thus, the influence of non-covalent contacts on the molecular structure, playing also a significant role within the real crystal structure, were not taken into account. Nevertheless, the calculated geometry is typical for DOTA-like compounds, which is highly suitable for a lanthanide complexation [1].

**Figure 7:**
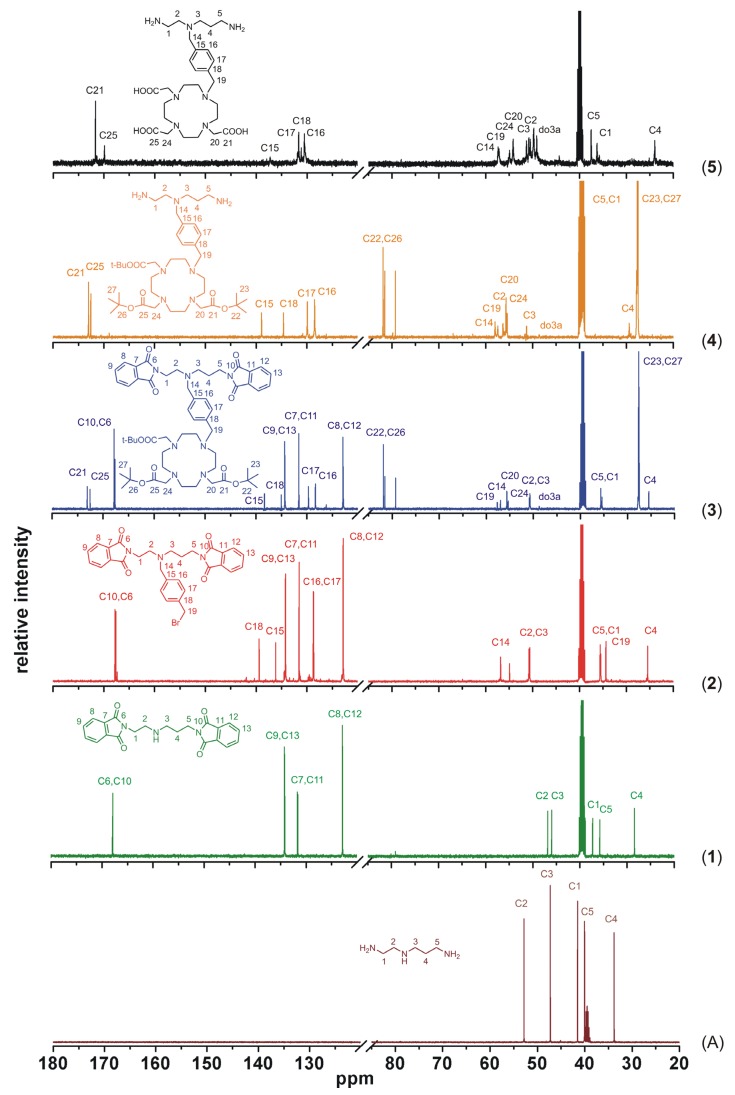
Comparison of ^13^C-NMR spectra of the precursor *N*-(2-aminoethyl)propane-1,3-diamine (**A**), intermediates **1**–**4** and the final product **5**, together with signals assignment (do3a represents unnumbered carbon atoms of the macrocycle).

To better illustrate the progress of the whole synthetic pathway, a comparison of ^13^C-NMR spectra of the precursor *N*-(2-aminoethyl)propane-1,3-diamine and all the described compounds **1**–**5** is shown in Figure 7. Based on the analysis of two-dimensional NMR spectra (selected spectra can be found in the Supplementary Materials Figures S1–S7), all signals in ^13^C-NMR spectra were assigned to the corresponding atoms, whose numbering is displayed in each spectrum in Figure 7, except for the macrocyclic carbon atoms (in the spectra labeled as do3a), which were not sufficiently resolved even at higher temperature. But such signal broadening is typical for this type of cyclen-based compounds and it usually complicates especially ^1^H-NMR spectra interpretation. Nevertheless, this ^13^C-NMR spectra comparison sufficiently documents each synthetic step because the presence of new signals corresponding to the new-bounded molecular parts (compounds **1**–**3**, synthetic steps i–iii in Scheme 1) and the absence of signals of the removed protecting groups (compounds **4** and **5**, synthetic steps iv and v in Scheme 1) are evident.

The described synthetic approach is relatively rapid, facile and provides pure products in good to moderate yields. The traces of acetic acid after the phthaloyl protection have to be removed otherwise undesired side-products are formed during the synthesis of **1**. Unfortunately, this operation significantly reduces the reaction yield. Especially, the yield of the synthesis of the intermediate **2** could be improved by increasing the excess of 1,4-bis(bromomethyl)benzene. Both phthaloyl and *t*-butyl protecting groups are easily and almost quantitatively removed providing the desired compounds, *i.e.*, **4**, and **5**, respectively. The prepared compound **5** itself may serve as a polydentate ligand involving the DO3A cavity suitable for complexation of lanthanide ions and a linear non-symmetrical triamine part for complexation of transition metal ions. Such a binuclear bimetallic system promises an interesting study of influence of the two metals and could provide combinations of different properties coming from different metals incorporated into a concrete complex (e.g., magnetic properties, spin-transition and luminescence). The xylylen spacer is not so common for linking DO3A with other molecules and it was proven that the xylylen substituent on the DO3A macrocycle has a positive effect on the lanthanide luminescence in the complex [14]. Moreover, the prepared compound scaffold is a versatile platform suitable for further structural modifications on the secondary amino groups. These secondary nitrogen atoms can be substituted with different pendant arms (acetate, methylphosphonate or methylenepyridine). This triamine part is also available for cyclization to produce a second macrocycle (possible different substituents) or a preparation of different types of Schiff bases can be taken into account as well. The described synthetic procedure is also applicable to *N*-(2-aminoethyl)ethane-1,3-diamine or *N*-(2-aminopropyl)propane-1,3-diamine instead of *N*-(2-aminoethyl)propane-1,3-diamine which would lead to more symmetrical species.

## 3. Experimental

### 3.1. General

Phthalic acid anhydride, *N*-(2-aminoethyl)propane-1,3-diamine, 1,4-bis(bromomethyl)benzene, hydrazine monohydrate, trifloroacetic acid and all solvents were purchased from commercial sources (Sigma–Aldrich, St. Louis, MO, USA; Penta, Prague, Czech Republic) and they were used as received. DO3A-tris(*tert*-butyl ester) hydrobromide was a gift from Dr. V. Kubíček from Charles University in Prague.

**Figure 8:**
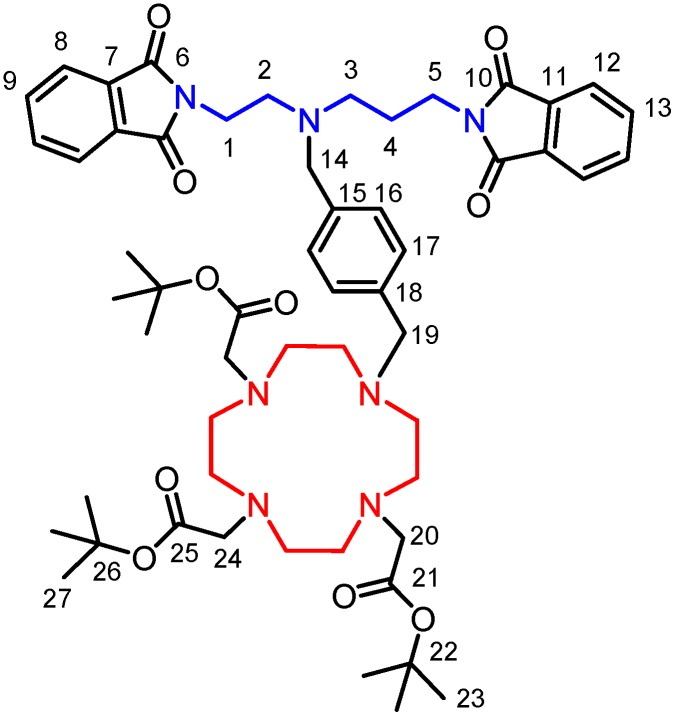
Schematic representation of the intermediate **3**, together with the atom numbering used for the interpretation of NMR spectra of all intermediates as well as the final product **5**.

^1^H- and ^13^C-NMR spectra were recorded on a 400 MHz NMR Varian spectrometer (Varian, Santa Clara, CA, USA) at 25 °C and 85 °C (^1^H-NMR spectra of compound **3**–**5**): ^1^H 399.95 MHz, dimethyl sulfoxide-*d*_6_ (DMSO, residual solvent peak) δ = 2.50 ppm, ^13^C 100.60 MHz, DMSO (residual solvent peak) δ = 39.51 ppm. Multiplicity of the signals is indicated as follows: s—singlet, d—doublet, t—triplet, qr—quartet, q—quintet, m—multiplet, br—broad. The deuterated solvent DMSO-*d*_6_ from Sigma Aldrich was used as received. The atom numbering scheme used for NMR data interpretation of all described compounds is shown in Figure 8.

The carbon as well as hydrogen atoms were assigned according to the spectra obtained from two-dimensional correlation experiments ^1^H–^1^H gs-COSY, ^1^H–^13^C gs-HMQC and ^1^H–^13^C gs-HMBS (gs = gradient selected, COSY = correlation spectroscopy, HMQC = heteronuclear multiple quantum coherence and HMBC = heteronuclear multiple bond coherence). Examples of two-dimensional NMR spectra can be found in Supplementary Materials (Figures S1–S7). Mass spectra were measured on an LCQ Fleet Ion Mass Trap mass spectrometer (Thermo Scientific, Waltham, MA, USA) equipped with an electrospray ion source and 3D ion-trap detector in the positive mode. Elemental analyses (C, H, N) were performed on a Flash 2000 CHNS-O Elemental Analyzer (Thermo Scientific). Elemental analysis for compounds **4** and **5** was not done due to its hydroscopic character. Merck (Darmstadt, Germany) aluminum foils with silica gel 60 F254 impregnated with a fluorescent dye were used for thin layer chromatography.

Theoretical calculations at the DFT level were used to optimize the geometry of the final product **5**. The calculations were performed with a Gaussian 03 program [26] based on the B3LYP functional with the 6-311G * basis set.

### 3.2. (2-Phthalimidoethyl)(3-phthalimidoprop-1-yl)amine (**1**)

Compound **1** was synthesized according to a slightly modified literature procedure [20,21]. The solid obtained after crystallization from EtOH (22.6 g) was dissolved in CHCl_3_ (200 mL) and extracted twice with 10% aqueous solution of Na_2_CO_3_, once with water and dried over anhydrous Na_2_SO_4_. The desired product was obtained after the evaporation of the solvent in a form of a pale yellow crystalline solid (15.1 g; 69% yield based on 7.04 g of *N*-(2-aminoethyl)propane-1,3-diamine). The compound **1a** was obtained after the evaporation of EtOH mother liquor and crystallization from MeOH (1.20 g).

(**1a**): NMR (DMSO): ^1^H δ 1.91 (H4, 2H, m); 2.99 (H3, 2H, m); 3.19 (H2, 2H, t, ^3^*J*_HH_ = 5.7 Hz); 3.63 (H5, 2H, t, ^3^*J*_HH_ = 6.7 Hz); 3.85 (H1, 2H, t, ^3^*J*_HH_ = 5.7 Hz); 7.49 (CH phthalate, 2H, m); 7.81–7.87 (H8, H9, H12, H13, 8H, m); 8.12 (CH phthalate, 2H, m); ^13^C{^1^H} δ 25.12 (C4, 1C, s); 34.44 (C5, 1C, s); 34.83 (C1, 1C, s); 44.74 (C3, 1C, s); 45.28 (C2, 1C, s); 123.03 (C12, 2C, s); 123.10 (C8, 2C, s); 130.30 (CH phthalate, 2C, s); 131.71 (C11, 2C, s); 131.85 (C6, 2C, s); 132.38 (C phthalate, 2C, s); 134.39 (C13, 2C, s); 134.43 (C9, 2C, s); 134.82 (CH phthalate, 2C, s); 167.93 (C10, 2C, s); 168.00 (C6, 2C, s); 168.15 (CO phthalate, 2C, s).

(**1**): NMR (DMSO): ^1^H δ 1.65 (H4, 2H, t, ^3^*J*_HH_ = 7.0 Hz); 1.86 (NH, 1H, bs); 2.52 (H3, 2H, t, ^3^*J*_HH_ = 7.0 Hz); 2.71 (H2, 2H, t, ^3^*J*_HH_ = 6.5 Hz); 3.57 (H5, 2H, t, ^3^*J*_HH_ = 7.0 Hz); 3.61 (H1, 2H, t, ^3^*J*_HH_ = 6.5 Hz); 7.80–7.85 (H8, H9, H12, H13, 8H, m); ^13^C{^1^H} δ 28.43 (C4, 1C, s); 35.78 (C5, 1C, s); 37.30 (C1, 1C, s); 46.00 (C3, 1C, s); 46.84 (C2, 1C, s); 122.85 (C12, 2C, s); 122.90 (C8, 2C, s); 131.62 (C11, 2C, s); 131.73 (C7, 2C, s); 134.19 (C13, 2C, s); 134.26 (C9, 2C, s); 167.90 (C10, 2C, s); 167.96 (C6, 2C, s); MS(+): *m/z* 378.25 [M+H]^+^, 400.25 [M+Na]^+^, 755.25 [2M+H]^+^; elemental analysis for C_21_H_19_N_3_O_4_, *M*_r_ = 377.39, found (calculated): C 66.93 (66.83); H 5.12 (5.07); N 11.09 (11.13).

### 3.3. (2-Phthalimidoethyl)(3-phthalimidoprop-1-yl)(4-bromomethylbenzyl)amine (**2**)

1,4-bis(Bromomethyl)benzene (4.5 g, 17.0 mmol, 2.2 eqv.) and anhydrous K_2_CO_3_ (6.10 g, 44.2 mmol, 5.6 eqv.) were placed in the two-neck 250 mL round bottom flask and MeCN (100 mL) was added. One neck was equipped with a spiral condenser and the second neck with a dropping funnel containing the solution of **1** (3.0 g, 7.9 mmol) in CHCl_3_ (20 mL). The mixture in the flask was heated to reflux under stirring and the solution was added dropwise during 30 min. The reaction was monitored by TLC (SiO_2_, ethyl acetate-heptane 1:1, *R_f_* = 0.5). For completion of the reaction, the mixture was refluxed for additional 30 min and then it was cooled down to room temperature. The solution was filtered through an S4 glass frit and the solvents were removed under reduced pressure. The crude product was purified by silica gel chromatography (DCHM–diethylether from 10:0 to 10:1). The desired compound was obtained after the evaporation of all solvents as a colorless solid (2.28 g, 51% yield). NMR (DMSO): ^1^H δ 1.72 (H4, 2H, q, ^3^*J*_HH_ = 7.0 Hz); 2.49 (H3, 2H, m); 2.58 (H2, 2H, t, ^3^*J*_HH_ = 5.7 Hz); 3.49 (H14, 2H, s); 3.55 (H5, 2H, t, ^3^*J*_HH_ = 7.0 Hz ); 3.62 (H1, 2H, t, ^3^*J*_HH_ = 5.9 Hz); 4.53 (H19, 2H, s); 7.05 (H16–H17, 4H, AA’BB’); 7.82 (H8,H9,H12,H13, 8H, m); ^13^C{^1^H} δ 25.45 (C4, 1C, s ); 34.36 (C19, 1C, s); 35.47 (C1, 1C, s); 35.60 (C5, 1C, s); 50.60 (C3, 1C, s); 50.77 (C2, 1C, s); 56.81 (C14, 1C, s); 122.89 (C8, C12, 4C, s); 128.68 (C17, 2C, s); 128.77 (C16, 2C, s); 131.57 (C7, C11, 4C, m); 134.25 (C9, C13, 4C, m); 136.16 (C15, 1C, s); 139.41 (C18, 1C, s); 167.65 (C6, 2C, s); 167.82 (C10, 2C, s); MS (+): *m/z* 560.18 [M+H]^+^, 584.08 [M+Na]^+^, 598.17 [M+K]^+^; elemental analysis for C_29_H_26_BrN_3_O_4_, *M*_r_ = 560.44, found (calculated): C 61.63 (62.15); H 4.72 (4.68); N 7.15 (7.50), Br 15.20 (14.26).

### 3.4. 1-{4-[(2-Phthalimidoethyl)(3-phthalimidoprop-1-yl)aminomethyl]phenylmethyl}-4,7,10-tris(t-butoxycarbonylmethyl)-1,4,7,10-tetraazacyclododecane (**3**)

Compound **3** was synthesized according to the modified literature procedure [27]. DO3A-tris(*t*-butyl ester) hydrobromide (1.59 g, 2.67 mmol) and anhydrous K_2_CO_3_ (1.94 g, 14.05 mmol, 5 eqv.) were placed into a 250 mL round bottom flask and MeCN (150 mL) was added. The mixture was heated to reflux under stirring. The solution of **2** (1.57 g, 2.81 mmol, 1.05 eqv.) in MeCN (20 mL) in a syringe was added dropwise in 5 min. The reaction mixture was refluxed for other 4 h and then it was cooled down to room temperature. The inorganic salts were filtered off on an S4 glass frit and the filtrate was evaporated under reduced pressure. The crude product was purified by silica gel column chromatography (CHCl_3_–MeOH = 25:1). The desired compound was obtained after the evaporation of the solvents as a light yellow solid foam (2.55 g, 96% yield). NMR (DMSO): ^1^H δ 1.42 (H23, 18H, s); 1.43 (H27, 9H, s); 1.74 (H4, 2H, q, ^3^*J*_HH_ = 7.2 Hz); 2.45 (CH_2_-do3a, 12H, bs); 2.52 (H3, 2H, t, ^3^*J*_HH_ = 7.2 Hz); 2.62 (H2, 2H, t, ^3^*J*_HH_ = 6.3 Hz); 3.01 (CH_2_-do3a, 4H, bs); 3.08 (H20, H24, 6H, m); 3.51 (H14, H19, 4H, bs); 3.55 (H5, 2H, t, ^3^*J*_HH_ = 7.2 Hz); 3.63 (H1, 2H, t, ^3^*J*_HH_ = 6.3 Hz); 7.07 (H16, H17, 4H, bs); 7.73–7.82 (H8, H9, H12, H13, 8H, m); ^13^C{^1^H} δ 25.47 (C4, 1C, s ); 27.53 (C27, 3C, s); 27.63 (C23, 6C, s); 35.43 (C1, 1C, s); 35.67 (C5, 1C, s); 48.76 (CH_2_-do3a, 8C, bs); 50.73 (C3, 1C, s); 50.86 (C2, 1C, s); 55.36 (C24, 1C, s); 55.64 (C20, 2C, s); 56.98 (C14, 1C, s); 57.67 (C19, 1C, s); 81.55 (C26, 1C, s); 81.86 (C22, 2C, s); 122.88 (C12, 2C, s); 122.95 (C8, 2C, s); 128.35 (C16, 2C, s); 129.71 (C17, 2C, s); 132.57 (C11, 2C, s); 131.67 (C7, 2C, s); 134.34 (C13, 2C, s); 134.36 (C9, 2C, s); 135.04 (C18, 1C, s); 138.34 (C15, 1C, s); 167.66 (C6, 2C, s); 167.86 (C10, 2C, s); 172.55 (C25, 1C, s); 173.14 (C21, 2C, s); MS(+): *m/z* 994.58 [M+H]^+^, 1016.58 [M+Na]^+^; elemental analysis for C_55_H_75_N_7_O_10_·1.6CHCl_3_, *M*_r_ = 1173.29, found (calculated): C 57.25(57.36); H 6.68 (6.51); N 8.30 (8.27).

### 3.5. 1-{4-[(2-Aminoethyl)(3-aminoprop-1-yl)aminomethyl]phenylmethyl}-4,7,10-tris(t-butoxycarbonyl-methyl)-1,4,7,10-tetraazacyclododecane (**4**)

The synthesis of **4** was done according to the literature procedure [23] with minor modifications. Absolute EtOH was not used. The rest of phthalhydrazide present in the residue was removed by filtration of the CHCl_3_ solution (the residue dissolved in 20 mL of CHCl_3_) through an S4 glass frit. The desired product was obtained as yellow oil (0.70 g, 95% yield based on 1.00 g of **3**). NMR (DMSO): ^1^H δ 1.46 (H23, H27, 27H, m); 1.55 (H4, 2H, q, ^3^*J*_HH_ = 6.8 Hz); 2.46 (H2, H3, 4H, m); 2.55 (CH_2_-do3a, 12H, bm); 2.60 (H1, H5, 4H, m); 3.08 (CH_2_-do3a, 4H, bs); 3.13 (H20, H24, 6H, bs); 3.53 (H14, 2H, s), 3.62 (H19, 2H, s); 7.27 (H16–17, 4H, AA’BB’); ^13^C{^1^H} δ 27.53 (C27, 3C, s); 27.64 (C23, 6C, s); 29.39 (C4, 1C, s); 39.26 (C1, 1C, s); 39.84 (C5, 1C, s); 48.58 (CH_2_-do3a, 4C, bs); 51.26 (C3, 1C, s); 51.60 (CH_2_-do3a, 4C, bs); 55.41 (C24, 1C, s); 55.65 (C20, 2C, s); 56.32 (C2, 1C, s); 57.43 (C19, 1C, s); 58.00 (C14, 1C, s); 81.52 (C26, 1C, s); 81.87 (C22, 2C, s); 128.50 (C16, 2C, s); 129.93 (C17, 2C, s); 134.63 (C18, 1C, s); 138.93 (C15, 1C, s); 172.56 (C25, 1C, s); 173.01 (C21, 2C, s); MS (+): *m/z* 734.58 [M+H]^+^, 756.58 [M+Na]^+^, 772.42 [M+K]^+^.

### 3.6. 1-{4-[(2-Aminoethyl)(3-aminoprop-1-yl)aminomethyl]phenylmethyl}-4,7,10-tris(carboxymethyl)-1,4,7,10-tetraazacyclododecane (**5**)

The deprotection of the *t*-butyl ester groups was done according to the literature procedure using TFA in DCHM [24]. The reactant **4** (0.70 g, 0.95 mmol) was dissolved in DCHM (5 mL) in a 50 mL round bottom flask and TFA (5 mL) was added dropwise under cooling to 0 °C in a water/ice bath. A slightly yellow solution was formed and additionally stirred at room temperature for 12 h. The solvents were removed *in vacuo*. The residue was purified on a cation-exchange column (Dowex 50, H^+^-form, 50 mL, elution with water and 15% aq. HCl) and an anion-exchange column (Dowex 1 × 8, OH^−^-form, 100 mL, elution with water and 15% aq. HCl). The solvent was removed under reduced pressure, and the residue was dissolved in MeOH. The desired compound was obtained after the evaporation of the MeOH solution as a yellow solid in the form of the corresponding hydrochloride (0.644 g, 90% yield).

NMR (DMSO): ^1^H δ 1.90 (H4, 2H, q, ^3^*J*_HH_ = 7.2 Hz); 2.76 (H3, 2H, t, ^3^*J*_HH_ = 7.2 Hz); 2.87 (H5, 2H, t, ^3^*J*_HH_ = 7.2 Hz); 2.93 (H2, 2H, t, ^3^*J*_HH_ = 6.8 Hz); 3.05 (H1, 2H, t, ^3^*J*_HH_ = 6.8 Hz); 3.10 (CH_2_-do3a, 4H, bs); 3.20 (CH_2_-do3a, 8H, m); 3.28 (CH_2_-do3a, 4H, bs); 3.44 (H20, 4H, bs); 3.87 (H14,H24, 4H, bs); 4.34 (H19, 2H, s); 7.38–7.56 (H16–17, 4H, AA’BB’); ^13^C{^1^H} δ 23.51 (C4, 1C, s); 35.82 (C1, 1C, s); 37.08 (C5, 1C, s); 48.70 (CH_2_-do3a, 2C, bs); 49.30 (CH_2_-do3a, 4C, bs); 50.08 (C2, 1C, s); 50.35 (C3, 1C, s); 50.87 (CH_2_-do3a, 2C, bs); 53.72 (C20, 2C, s); 54.47 (C24, 1C, s); 56.71 (C19, 1C, s); 56.93 (C14, 1C, s); 130.08 (C16, 2C, s); 130.70 (C18, 2C, s); 131.19 (C17, 2C, s); 136.83 (C15, 1C, s); 169.44 (C25, 1C, s); 171.22 (C21, 2C, s); MS (+): *m/z* 566.50 [M+H]^+^; MS^2^ (+): *m/z* 548.22 [M+H–H_2_O]^+^, 530.42 [M+H–2H_2_O]^+^, 512.42 [M+H–3H_2_O]^+^.

## 4. Conclusions

The compound 1-{4-[(2-aminoethyl)(3-aminoprop-1-yl)aminomethyl]phenylmethyl}-4,7,10-tris-(carboxymethyl)-1,4,7,10-tetraazacyclododecane (**5**) was prepared and characterized with the aim to design a molecule which could be usable as a versatile polydentate ligand in the field of coordination chemistry. The molecule combines a macrocyclic part which could be suitable for the complexation of lanthanides and linear amine part usable for the complexation of other (transition) metal ions. All the intermediates as well as final product were thoroughly characterized. The reaction conditions for the preparation of the intermediate **2** were investigated and optimized, while the other synthetic steps proceeded according to the slightly modified previously described literature procedures. The xylylen linker was chosen for the connection of the polyamino and DO3A parts due to its rigidity and short length providing a possible interaction between the two metal centers in a bimetallic complex, good synthetic availability. Surprisingly, such a motif is not so common in the literature. Altogether, the prepared compound **5** represents a versatile ligand with at least two sites of possible coordination of a lanthanide and transition metal. Further structural modifications on secondary amino groups are straightforward and could provide an additional possibility of the system transformation/tuning.

## Supplementary Materials

Supplementary materials, containing 2D NMR spectra of **1**–**5** (Figures S1–S7), can be accessed at: http://www.mdpi.com/1420-3049/18/11/13940/s1.
