# Supplementary material for: Synthesis of a Versatile Building Block Combining Cyclen-derivative DO3A with a Polyamine via a Rigid Spacer
Source: Molecules. 2013 Nov 12;18(11):13940–56. doi: 10.3390/molecules181113940 (PMC6269841; doi:10.3390/molecules181113940)

Supplementary Materials

Figure S1. Partial ^{1}H–^{13}C gs-HMQC NMR spectrum of **1**, (2-phthalimidoethyl)(3-phthalimidoprop-1-yl)amine, (DMSO-d_6) with a residual peak of H_2O at 3.32 ppm.

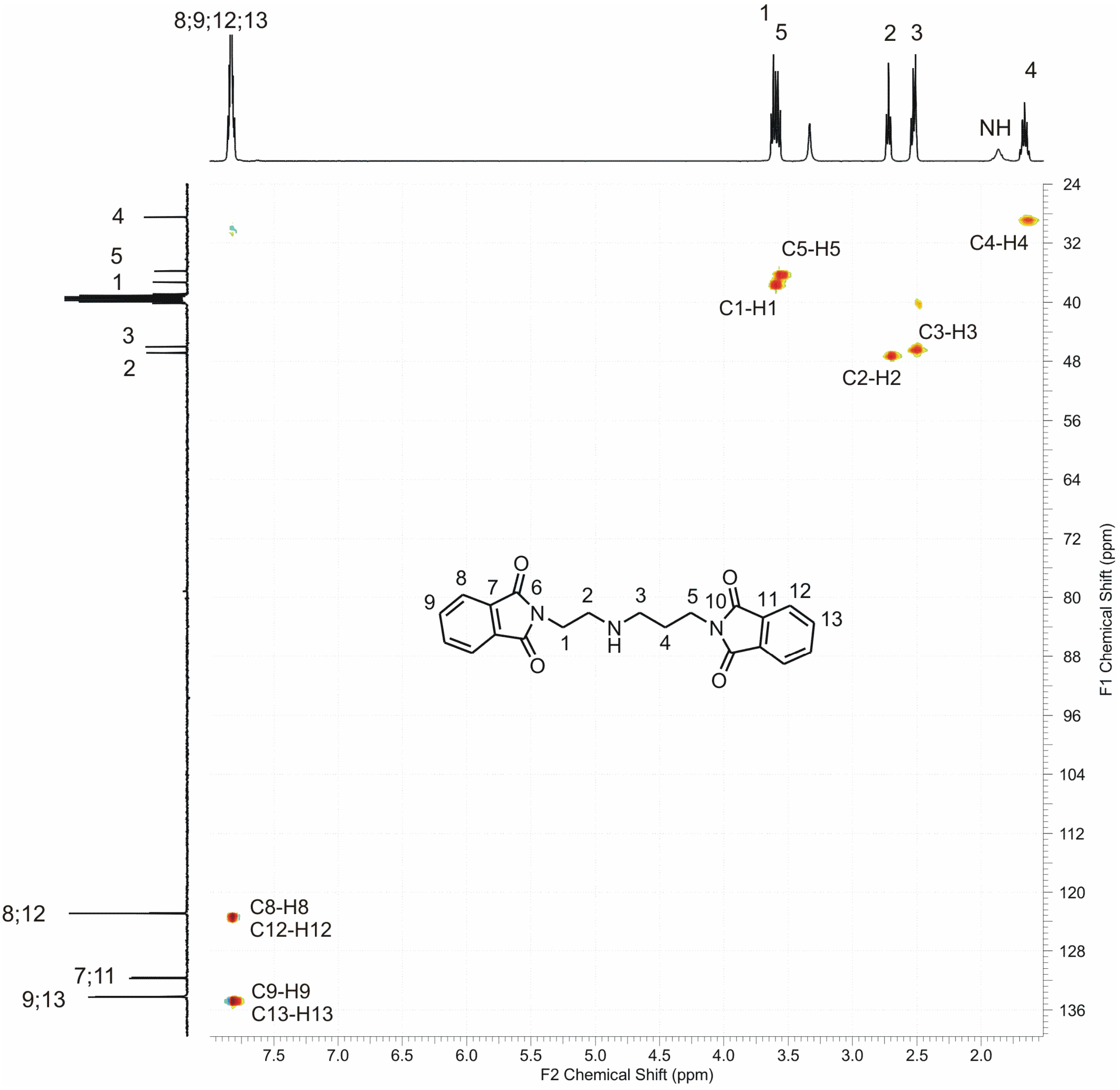

Figure S2. Partial ^{1}H–^{13}C gs-HMQC NMR spectrum of **2**, (2-phthalimidoethyl)(3-phthalimidoprop-1-yl)-(4-bromomethylbenzyl)amine, (DMSO-d_6) with a residual peak of H_2O at 3.34 ppm and CH_2Cl_2 at 5.75 ppm.

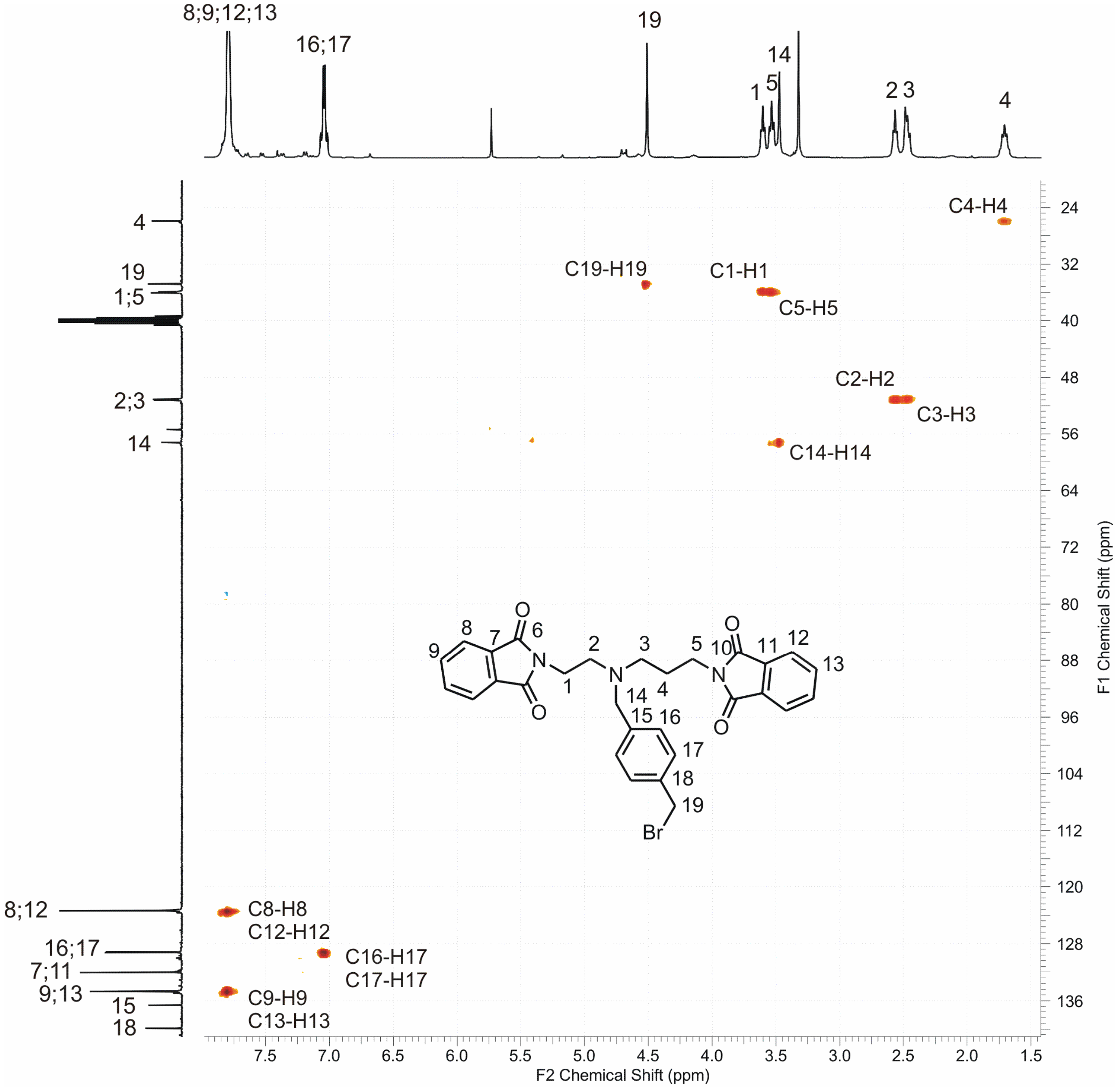

Figure S3. Partial ^{1}H–^{13}C gs-HMQC NMR spectrum of **3**, 1-{4-[(2-phthalimidoethyl)(3-phthalimidoprop-1-yl)aminomethyl]phenylmethyl}-4,7,10-tris(*tert*-butoxycarbonylmethyl)-1,4,7,10-tetraazacyclododecane, (DMSO-d_6) with a residual peak of H_2O at 3.40 ppm.

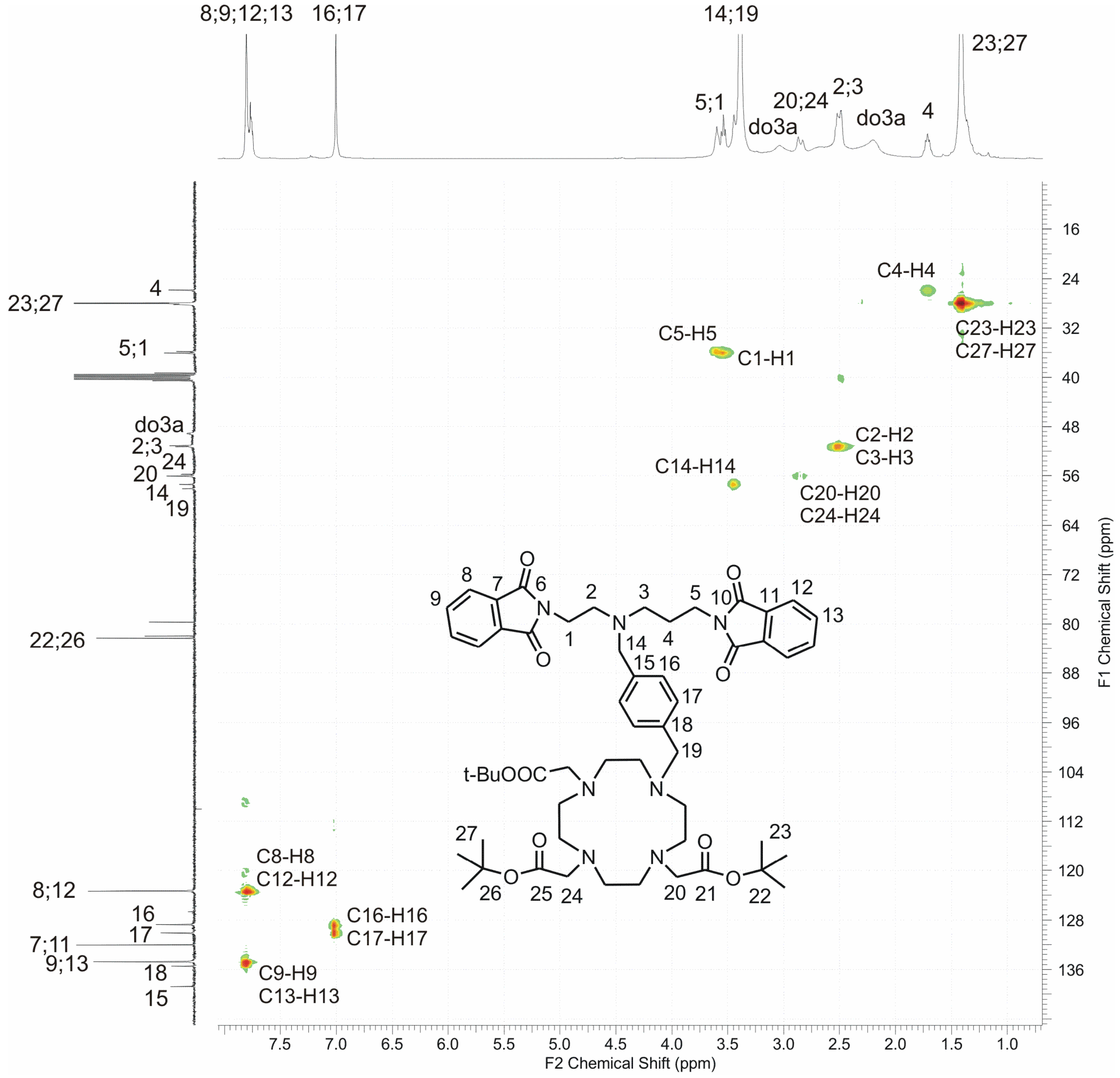

Figure S4. Partial ^{1}H–^{13}C gs-HMQC NMR spectrum of **4**, 1-{4-[(2-aminoethyl)(3-aminoprop-1-yl)aminomethyl]phenylmethyl}-4,7,10-tris(*tert*-butoxycarbonylmethyl)-1,4,7,10-tetraazacyclododecane, (DMSO-d_6) with a residual peak of H_2O at 3.40 ppm.

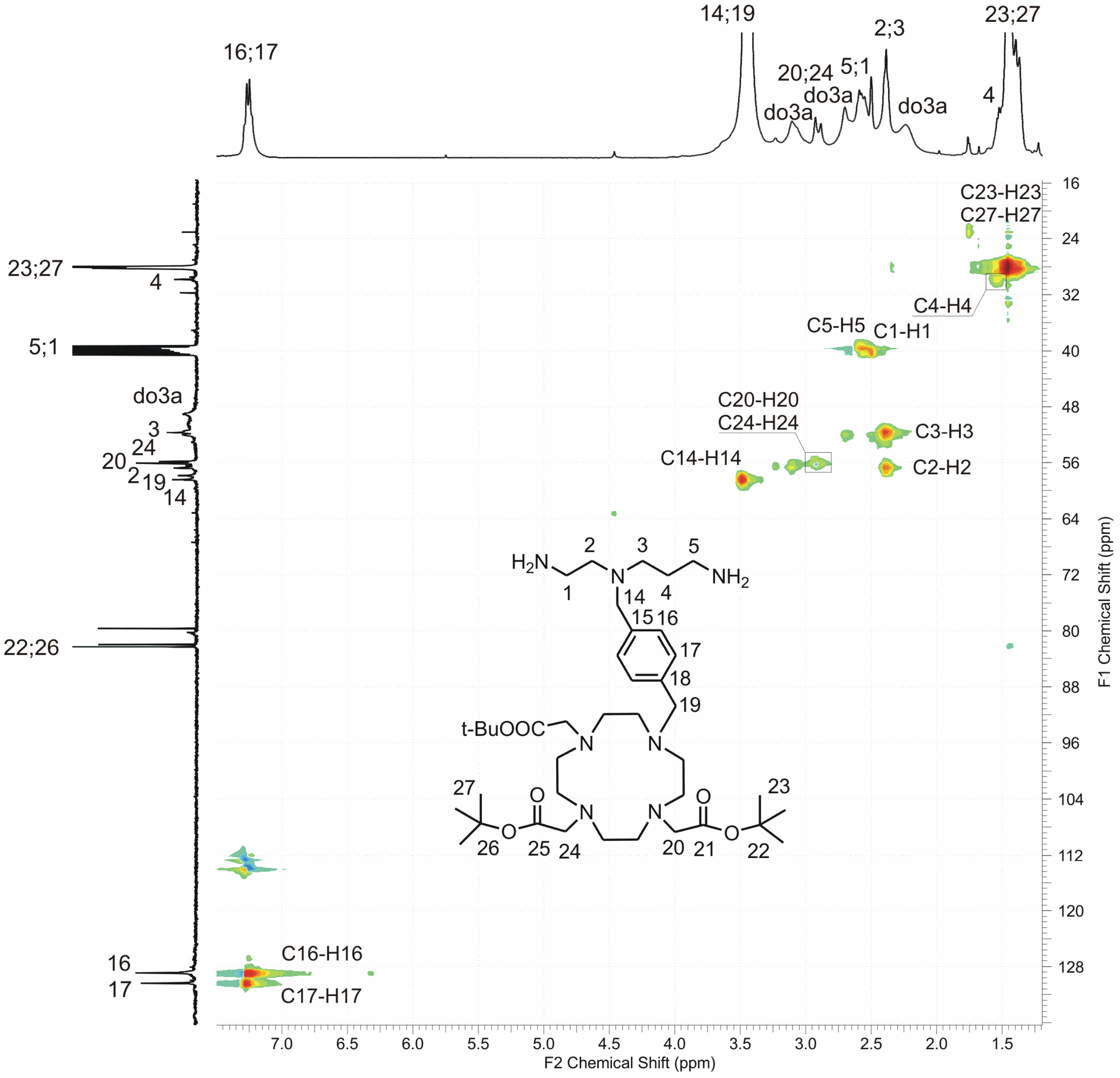

Figure S5. Partial ^{1}H–^{13}C gs-HMQC NMR spectrum of **5**, 1-{4-[(2-aminoethyl)(3-aminoprop-1-yl)aminomethyl]phenylmethyl}-4,7,10-tris(carboxymethyl)-1,4,7,10-tetraazacyclododecane, (DMSO-d_6).

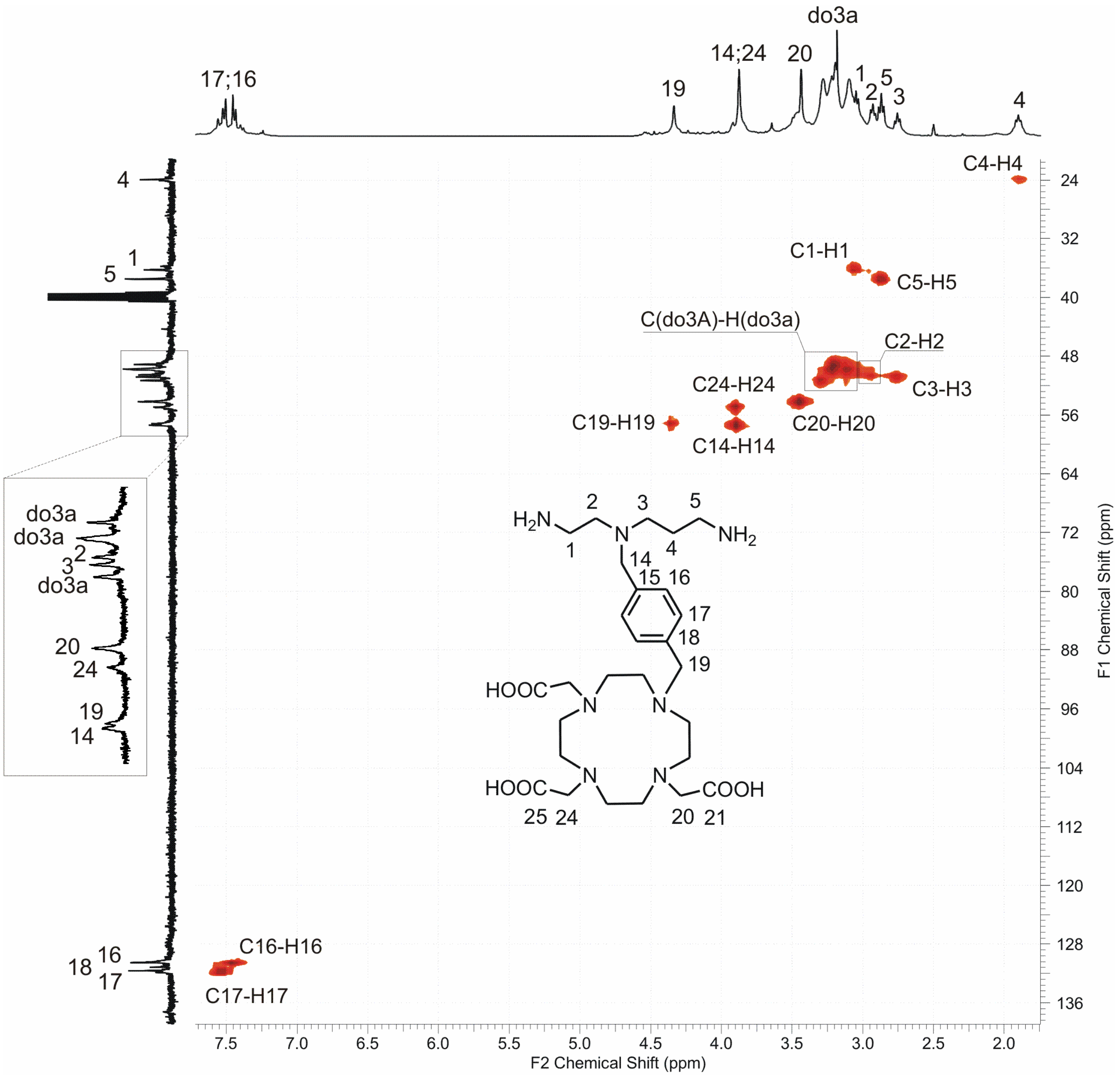

Figure S6. Partial ^{1}H–^{13}C gs-HMBC NMR spectrum of **5**, 1-{4-[(2-aminoethyl)(3-aminoprop-1-yl)aminomethyl]phenylmethyl}-4,7,10-tris(carboxymethyl)-1,4,7,10-tetraazacyclododecane, (DMSO-d_6).

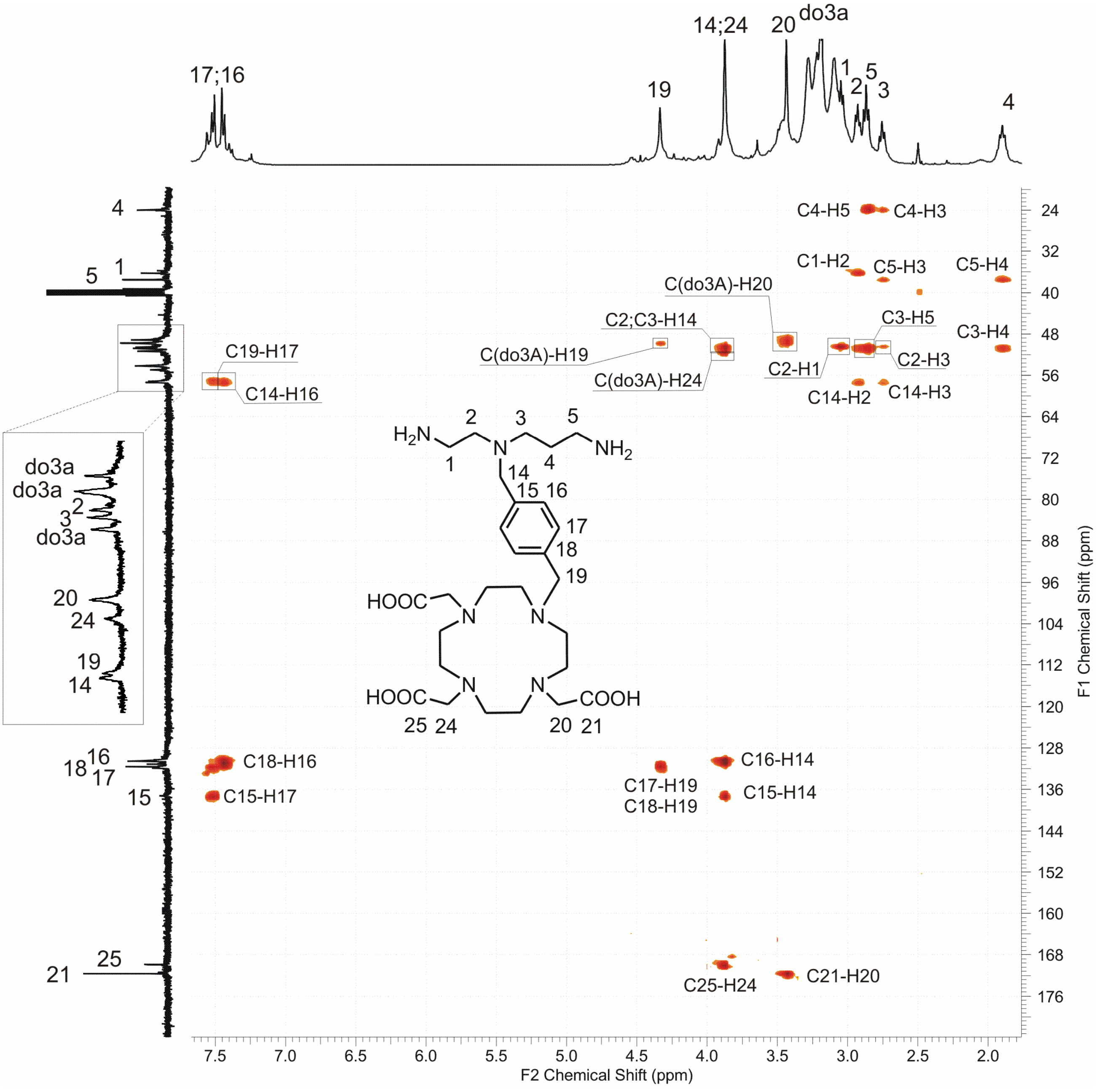

Figure S7. Partial ^{1}H–^{13}C gs-HMBC NMR spectrum of a precursor, *N*-(2-aminoethyl)propane-1,3-diamine (DMSO-d_6).

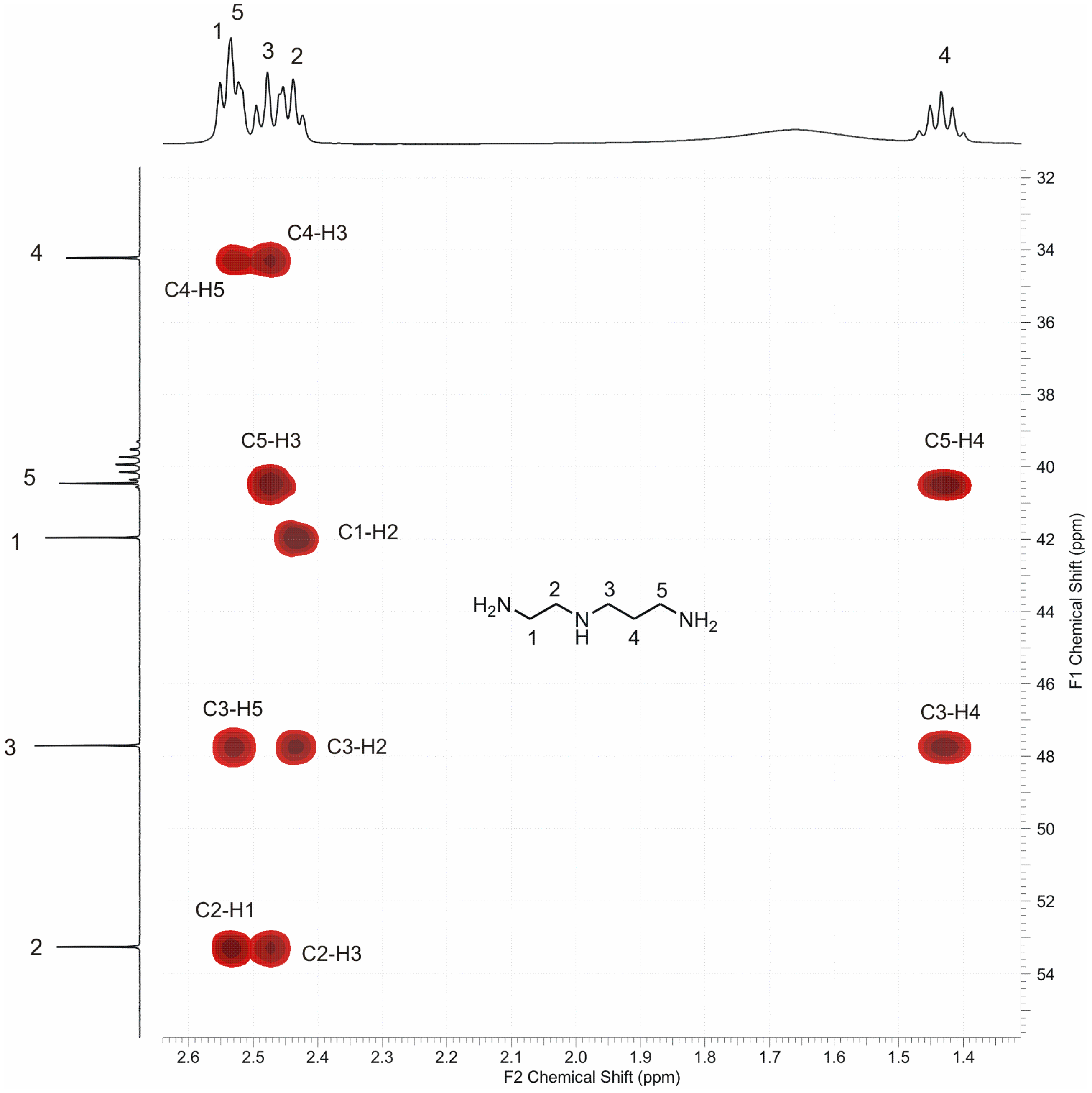

Supplement: Supplementary file 1 [file molecules-18-13940-s001.pdf]
